# Interplay of neutrophils and type I interferons in the development of end-organ damage in SLE

**DOI:** 10.1186/ar3968

**Published:** 2012-09-27

**Authors:** MJ Kaplan

**Affiliations:** 1 University of Michigan, Ann Arbor, MI, USA

## 

Patients with systemic lupus erythematosus (SLE) have up to a 50-fold increased risk of developing atherosclerotic cardiovascular disease that cannot be explained by the Framingham risk equation. While both SLE-specific and nonspecific mechanisms have been proposed to play a prominent role in the induction of premature vascular damage in this disease, the exact etiology remains unclear. We have proposed that an imbalance between vascular damage and repair probably induced by IFNα and other type I interferons could play a prominent role in the induction of accelerated atherosclerosis in SLE. Our group and others have recently elucidated the potential role that these cytokines play in the development and progression of premature atherosclerotic disease in SLE and, potentially, in other autoimmune diseases. More recently, a novel role for aberrant neutrophils in the development of autoimmune responses and vascular damage in SLE and other diseases has emerged; in particular, the role that neutrophil extracellular traps may play in the development of this disease and its vascular complications such as endothelial dysfunction and atherothrombosis. The recent description of a distinct subset of proinflammatory neutrophils isolated from lupus patients that induces vascular damage and synthesizes type I interferons has shed new light into a potentially important cell subset implicated in endothelial damage. Finally, novel discoveries pertaining to the interactions of lipoproteins and the immune system may prove quite informative in understanding how premature atherosclerotic plaque develops in lupus.

